# What explains between-school differences in rates of smoking?

**DOI:** 10.1186/1471-2458-8-218

**Published:** 2008-06-20

**Authors:** Marion Henderson, Russell Ecob, Daniel Wight, Charles Abraham

**Affiliations:** 1 MRC Social and Public Health Sciences Unit, Glasgow, UK; 2 Ecob Consulting, Glasgow, UK; 3 Department of Psychology, University of Sussex, Brighton, UK

## Abstract

**Background:**

Schools have the potential to influence their pupils' behaviour through the school's social organisation and culture (non-formal school characteristics), as well as through the formal curriculum. This paper examines whether these school characteristics (which include a measure of quality of social relationships) can account for school differences in smoking rates.

**Methods:**

This study uses a longitudinal survey involving 5,092 pupils in 24 Scottish schools. Pupils' smoking (at age 15/16), cognitive measures, attitude to school and pupils' rating of teacher pupil relationships (at age 13/14) were linked to school level data comprising teacher assessed quality of pupil-staff relationships, school level deprivation, staying on rates and attendance. Analysis involved multi-level modelling.

**Results:**

Overall, 25% of males and 39% of females reported smoking, with rates by school ranging from 8% to 33% for males and from 28% to 49% for females. When individual socio-economic and socio-cultural factors were controlled for there was still a large school effect for males and a smaller (but correlated) school effect for females at 15/16 years. For girls their school effect was explained by their rating of teacher-pupil relationships and attitude to school. These variables were also significant in predicting smoking among boys. However, the school effect for boys was most radically attenuated and became insignificant when the interaction between poor quality of teacher – pupil relationships and school level affluence was fitted, explaining 82% of the variance between schools. In addition, researchers' rating of the schools' focus on caring and inclusiveness was also significantly associated with both male and female smoking rates.

**Conclusion:**

School-level characteristics have an impact on male and female pupils' rates of smoking up to 15/16 years of age. The size of the school effect is greater for males at this age. The social environment of schools, in particular the quality of teacher-pupil relationships, pupils' attitude to school and the school's focus on caring and inclusiveness, can influence both boys' and girls' smoking. This provides support for the school-wide or "Health Promoting School" approach to smoking prevention.

## Background

Smoking is the biggest single cause of preventable death in the UK, causing 120,000 premature deaths each year, including a third of all cancer deaths. Consequently, the UK Chief Medical Officer's number 1, "tip for better health" is "Don't smoke and don't breathe others' tobacco smoke". [1,2] Most smokers begin smoking in adolescence and the earlier smoking begins, the harder it is to give up. [3] Moreover, decreases in adult smoking evident since the 1970s have not been matched amongst adolescent smokers [4,5] despite a government target to reduce the proportion of 11–15 year olds who smoke from 13% in 1996 to 9% by 2010 [6].

There is a growing consensus that school-based interventions are largely ineffective. [7,8] A contributing factor may be that, despite UK government policy emphasising social influences on smoking, [6] most research into the antecedents of adolescent smoking, and most intervention programmes, focus on individual characteristics rather than the environment in which adolescents smoke. A notable exception is the ASSIST programme, which recruits and trains school peer leaders to discourage uptake of smoking [9], currently being evaluated through a cluster randomised trial. Research on school-related, though not environmental, factors associated with adolescent smoking suggests that school performance [10] and self-perceived scholastic competence [11] are negatively associated with smoking. Moreover, smoking may provide a coping mechanism to stress [12] which may be more attractive to pupils who see themselves as struggling with the demands of school. [13] Such interactions between individual characteristics and the school-related factors highlight potential intervention targets but do not take account of underlying effects of the school environment itself. This study explores the extent to which school environments directly shape smoking behaviour.

The ideal of a Health Promoting School (HPS) currently guides school health policies and practice internationally [14,15], recommending that schools go beyond formal health education curricula to adopt school-wide health promoting practices which alter the quality of social relationships at an institutional level. Yet there is little empirical research into links between school characteristics and pupils' health behaviours. Evidence of such a link would support HPS recommendations and provide a powerful incentive for changing specified school characteristics.

The impacts of the institutional features of a school on the health-related behaviours of its pupils are called 'school effects', or school level variance. School effects are found when 'pupil outcomes for a school vary, either positively or negatively, from that which might be expected, given the known predictors of these outcomes.' [16] To date there have been two main studies of school effects on health-related behaviours in Britain. Aveyard et al. [17,18] found school effects on smoking for 15 – 16 year olds, but they were weaker than those found for younger age groups in the same schools. School effects were not analysed by gender and the study did not try and 'unpack' which features of the schools contributed to the school effects. West et al. also found school effects on smoking, as well as drinking and drug use, at 13 and 15 years, and for diet at 13 years only. [19] As with Aveyard et al.'s study, school effects were found to be stronger earlier on in secondary education. [19] In terms of school processes, West et al. (2004) found that school level smoking varied according to the degree of engagement (and involvement) of pupils with education and the number of teachers they got on with. Smoking was also associated with larger schools and those rated by researchers to have poorer ethos: together these variables explained the school effect. Again, these findings were not analysed by gender.

Aveyard et al and West et al. make three important methodological points. First, multi-level modelling should be used in order to take account of the hierarchical structure (or clustering) of these data, that is, pupils are nested within schools. Single level (OLS) regression techniques over-estimate school-effects due to underestimation of the standard errors. [19] Second, models should include known predictors, particularly socio-economic and family variables, but not those likely to result from attendance at the same school (e.g. peer smoking, since peers are likely to be exposed to the same school influence). Third, studies should adjust for baseline smoking status as this is likely to be the strongest predictor of subsequent smoking. [17,19] This study was designed to take account of these three points.

The aim of this study is to quantify and characterise school effects on smoking. Data from a randomised controlled trial of sex education in schools [20] are used to examine: a) which factors are significantly associated with smoking at average age 16 years, b) whether there are 'school effects' influencing the proportion of pupils reporting smoking by age 16, and c) if so, whether such 'school effects' can be attributed to identifiable school characteristics such as quality of relationships and attitudes towards school.

The study extends previous research by exploring school effects separately for each gender. In addition, the paper also aims to go further in 'unpacking' the school effects by adjusting for pupil and teacher perceived quality of relationships and the extent to which the school was judged to focus on caring and inclusiveness. This complements and adds to the dimensions studied by West et al. for their younger age groups (2004).

## Methods

Ethical permission for the intervention, an evaluation in the form of a cluster randomised trial, interviews with and questionnaires for both teachers and pupils was granted by Glasgow University's Ethical Committee for Non-Clinical Research Involving Human Subjects. Teachers and pupils were informed in detail about the research and provided their informed consent prior to involvement in the study. In addition, pupils' parents were also fully informed about the research and the parents had the opportunity to withdraw their children prior to the intervention and research commencing. [20] The trial complied with the Helsinki Declaration and was logged with the International Standard Randomised Controlled Trial Number Register and its registration number is ISRCTN48719575. [21]

The randomised control trial (RCT) of school sex education *(SHARE) *was conducted in non-denominational state schools within 15 miles of the main cities in Tayside and Lothian regions of Scotland. Out of 47 schools, 25 agreed to participate. One of the participating schools did not provide baseline data and so has been excluded from this analysis (School 4).

### Pupil level data

**Figure 1:**
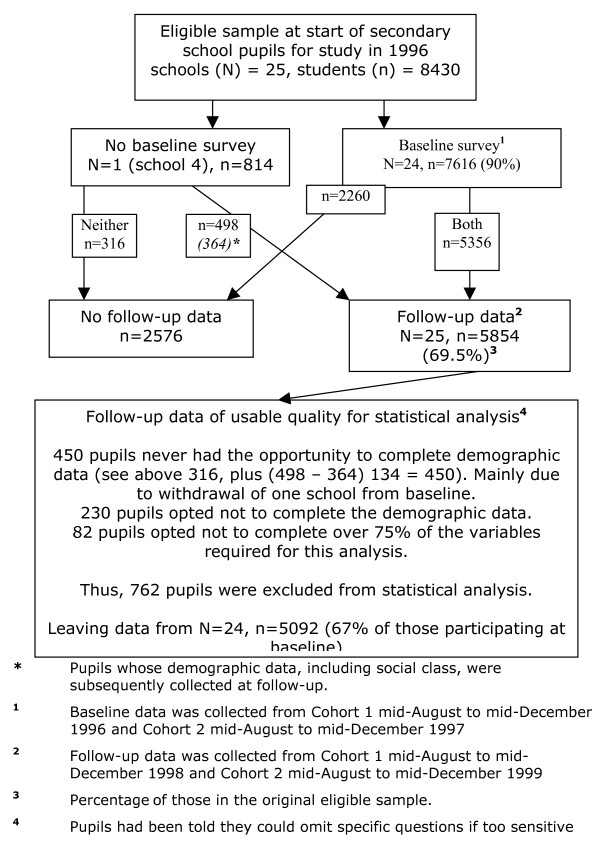
**Flowchart of participants.** N gives number of schools, and n number of pupils.

During 1996 and 1997 two successive cohorts of 13 – 14 year olds participated in a baseline survey (mean age 14 years and two months). These 7,616 pupils who participated (of the 8,430 eligible) were representative of 14 year olds throughout Scotland, in terms of parents' social class and the proportion of one-parent households, using 1991 Census data. [22] This paper also uses data collected in 1998 and 1999 at the first follow-up of the SHARE trial, when the cohorts were aged 15 or 16 (mean age 16 years and one month). By this age 27% had left school. Follow-up data were collected from 5,854 young people giving an overall participation rate of 69.5% (see also Figure 1). There was a very different participation rate for those still at school (81%) and early school leavers (39%). A small proportion (2%) refused to participate (Wight et al., 2002). Early school leavers are those that leave as soon as they are legally old enough (16 years of age), which generally prevents them entering higher education, such as university or college.

For pupils at school, questionnaires were administered by trained researchers in classrooms under exam conditions with no teachers present. Questionnaires were sealed in envelopes on completion and had identity numbers, but not respondents' names. Those who had left school were contacted at home by post. The baseline questionnaire included questions on: pupils' smoking; socio-cultural variables (e.g. family composition and parental monitoring); and attitudinal variables (e.g. attitude to school and self-esteem). The pupils' questionnaires at baseline and age 16 follow-up broadly asked the same questions, although the age 16 questionnaire was slightly longer. The questionnaires (and additional information about *SHARE*) are available on a public domain Internet site. [23]

Pupils with no baseline or no demography data subsequently collected at follow-up (680 pupils) or with over 75% missing data on variables required for analyses (82 pupils) were excluded (they had been told they could omit specific questions if too sensitive). This generated a sample of 5,092 school pupils (67% of those who participated at baseline). Each model compared in this study includes the same 5,092 pupils. Descriptive statistics on the pupil level variables used in the analysis and how we handled missing data is provided in Table 1.

**Table 1:** Distribution of pupil level variables (all pupil level analyses involved 5,092 cases, except for the early school leavers' sub-group analysis as described in the text).

	**Descriptive Statistics as appropriate to scale of measurement**	**Missing data N (%)**	**How missing data was handled**	**Possible reason for the missing data**
Gender	47% male	0 (0)	N/A	N/A
Age in months at time of first interview	Mean = 169.8, sd = 3.9	471 (9.2)	Mean substitution by school and by gender	Some pupils were wary of providing their data of birth (necessary to calculate the age at time of interview) as they believed the answer could potentially be used to track their identity, thus they opted to leave it blank. Naturally, we had explained that we were not interested their identities.
Cohort (there were two cohorts)	52% belonged to cohort 1	0 (0)	N/A	N/A
Early school leavers	88% of pupils stayed on at school beyond the minimum legal age for attending school (16 years)	0 (0)	N/A	N/A
Family structure	75% lived with both parents, 21% with Mum only, 3% with Father only and 1% with neither parent.	0 (0)	N/A	N/A
Housing type	69% of pupils lived in privately owned housing, 22% rented housing and 0.2% were in care or foster homes	469 (9.2)	A separate category was coded for missing data	Some pupils were unsure if they lived in privately owned housing or not.
Parental monitoring (high vs low)	60% of pupils experienced high parental monitoring	0 (0)	N/A	N/A
Father occupational class (manual vs non-manual)	35% worked in manual occupations	1199 (23.5)	A separate category was coded for missing data	Some pupils found the questions about parental occupation intrusive and so left them blank. Others provided textual information that we did not find possible to classify.
Spending money (high vs low)	51% had over £15 spending money (high)	20 (0.4)	N/A	A few pupils did not get a consistent amount of spending money and felt they could not complete this question.
Ethnicity	96% of the pupils were 'white'	51 (1.0)	A separate category was coded for missing data	Some pupils did not see the point in the question and objected by leaving it blank
Mothers' age (<40 vs older mother)	49% had a mother over 40 years	720 (14.1)	A separate category was coded for missing data	Some pupils did not know their Mum's age and some did not see the point of the question and objected by leaving it blank (despite us explaining why we asked, whenever the issue was drawn to our attention).
Religious belief (5 pt scale, higher score less religious)	Mean = 3.9, sd = 1.0	0 (0)	N/A	N/A
Self-esteem (4 pt scale, higher score lower self-esteem)	Mean = 2.0, sd = 0.5	25 (0.5)	Mean substitution by school and by gender	Some pupils did not like there being no 'unsure' but that is the standard way to ask the questions.
Attitude to school (5 pt scale, higher score poorer attitudes)	Mean = 2.6, sd = 1.0	26 (0.5)	Mean substitution by school and by gender	No clear reason
Teacher-pupil relationships (5 pt scale, higher score poorer relationships)	Mean = 2.8, sd = 0.9	60 (1.2)	Mean substitution by school and by gender	No clear reason

The dependent measure of current smoking was a binary indicator of self-reported (regular or occasional) smoking at follow-up. Pupils were asked 'Have you tried, or do you use, any of the following?' including 'tobacco (cigarettes)'. They could select 'never tried', 'tried', 'use occasionally' or 'use regularly'. Those that ticked either 'use occasionally' or 'use regularly' were coded as (regular or occasional) smokers and the others were coded as non smokers.

### Data on school characteristics

**Figure 2:**
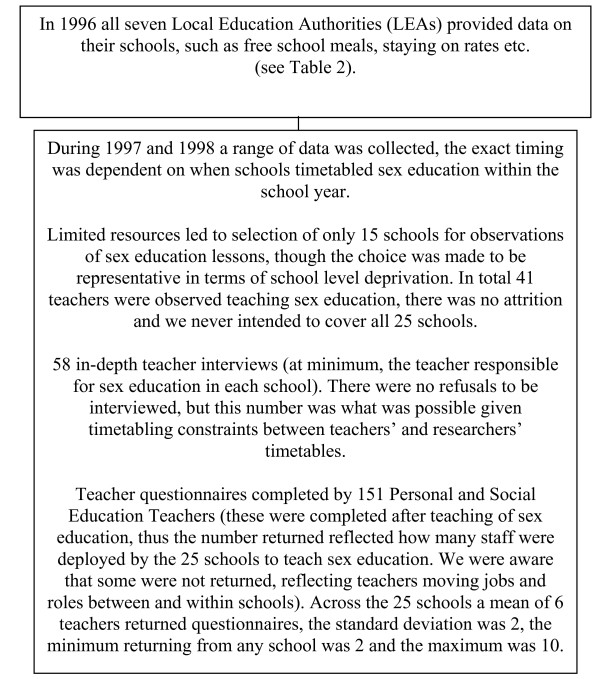
Data on school level characteristics collected between 1996 and 1998.

Data on school characteristics were collected using four methods (see also Figure 2). First, the pupil questionnaires (PQ), described above, asked about whether pupils liked school (2 items), and thought teachers trusted and respected them (2 items). All the PQ data were built in to the multi-level modelling at individual level. Second, information from 151 Personal and Social Education (PSE) teachers' questionnaires (TQ) reporting their perceptions of: senior management's attitude towards staff relationships (1 item); staff-staff relationships (2 items), and staff-pupil relationships (2 items), the answers for each school's TQs were averaged and entered into a factor analysis (described below) of school level information. Third, this factor analysis also included measures based on school level information provided by Local Education Authorities (LEAs) originally collected for balanced randomisation, i.e. proportion of free school meals, staying on rates, attendance, placing requests (for children to attend the school from parents living outwith the catchment area) and school size.[24,25] Descriptive statistics on the school level variables used in the analysis is provided in Table 2.

**Table 2:** Distribution of school level variables (all analyses involving school level variables included data from all 24 schools)

	**Descriptive Statistics as appropriate to scale of measurement**	**Missing data N (%)**	**How missing data was handled**	**Possible reason for the missing data**
Deprivation score of local area (linkage between school catchment area postcodes and Carstairs Index of Deprivation. The minimum score was -5 and maximum was 4, the higher the score the more affluent the school.)	Mean = -1.33, sd = 2.1	0 (0)	N/A	N/A
Employment in school catchment area	Mean = 10.5% unemployed, sd = 4.3%	0 (0)	N/A	N/A
Staying on rates from Secondary (S)4 to S5 (a score of 1 would mean everyone stayed on, so 0.77 equates to 77% staying on)	Mean = 0.77, sd = 0.09	0 (0)	N/A	N/A
Pupils' post school destination (mean reflects those gone to higher education or a job)	Mean = 72%, sd = 10.2	0 (0)	N/A	N/A
Free school meals	Mean = 12% free meals, sd = 8.8	0 (0)	N/A	N/A
School attendance	Mean = 90% attendance, sd = 2.7	0 (0)	N/A	N/A
Staying on rates from S5 to S6 (a score of 1 would mean everyone stayed on, so 0.59 equates to 59% staying on)	Mean = 0.59, sd = 0.16	0 (0)	N/A	N/A
Access to sexual health services (The higher the score the better the access to clinics in terms of transport links from school postcodes to clinic postcodes and in terms of access to dedicated youth provision. The range minimum score was 0 and maximum was 43)	Mean = 17.5, sd = 10.1	0 (0)	N/A	N/A
Parental placing requests for their child to attend a school (distinguishes urban from rural)	Mean = 13% parents requested school placing, sd = 11.9	0 (0)	N/A	N/A
Proportion of pupils from ethnic minority groups	Mean = 93% white, sd = 0.5	0 (0)	N/A	N/A
Quality of teacher relationships with each other (5 pt scale, the higher the score the poorer the quality)	Mean = 2.0, sd = 0.5	0 (0)	N/A	N/A
Quality of teacher-pupil relationships as rated by teachers (5 pt scale, the higher the score the poorer the quality)	Mean = 2.2, sd = 0.5	0 (0)	N/A	N/A
Size of the school (total number of pupils in S3 & S4 – the school years that *SHARE *would be taught)	Mean = 368 puils, sd = 82.7	0 (0)	N/A	N/A
Quality of sex education at the school (the higher the score the better the quality of sex education in terms of teacher training, quality of teaching materials and time allocated to the topic. The range minimum was 11.2 and maximum was 26.6)	Mean = 25.1, sd = 7.2	0 (0)	N/A	N/A

The fourth kind of information on school characteristics is qualitative, arising from 58 in-depth teacher interviews (at minimum, the teacher responsible for sex education in each school), observations of lessons with 41 teachers (in 15 out of the 24 schools) and numerous ethnographic notes (from all schools). These methods allowed most schools to be scored according to academic focus, on a scale of 1 (focus on academic achievement), 2 (in-between) and 3 (focus on caring and inclusiveness). Two qualitative researchers (including DW) coded the data and then reviewed all the relevant information, giving general scores for different dimensions of school culture. A second researcher validated this scoring and any discrepancies were discussed until a consensus score was agreed. It should be noted that the quantity and type of information held about each school varied considerably, and there were missing data for five schools. This is because the data collected was qualitative and was never intended to be systematically collected from each school. However, despite the limitations with the data and given the relevance of the data, it was decided that using the data as far as it was possible would add richness to this paper.

### Statistical analysis

A series of predictive models were fitted to pupils' smoking status (see Table 3). These allowed examination of results after adjustment for pupil-level and school-level characteristics. Comparing the variance due to school effects between models revealed which factors contributed to differences between schools.

**Table 3:** Sequences of models tested

**Model 1**	This model controlled for socio-demographic and cultural factors predicting smoking. Any 'school effect' can only be identified after adjustment for such factors.
	The variables included were; pupils' gender, age in months at follow-up, cohort, whether left school at youngest possible legal age, composition of the family unit in which pupils lived, housing tenure, levels of parental monitoring, father's social class, amount of personal spending money, ethnic group, and mother's age. Only one interaction with gender was found and added to the model – gender and living with mother only. All PQ data.
**Model 2**	This model adds (to Model 1) measures of self esteem and religiosity that may be influenced by school (but are likely to be more influenced by home environment). All PQ data.
**Model 3**	This model adds (to Model 2) cognitions related to school, that is, pupils' attitude to school and pupil-assessed teacher-pupil relationships. It was expected that these cognitions would be influenced by school experience (though, of course, home environment may also have been important). All PQ data
**Model 4**	This model adds (to Model 3) school level affluence (factor 1 – LEA data).
**Model 5**	This model adds (to Model 3) teacher-assessed school-level (poor) quality of relationships (both staff-staff and staff-pupil relationships – factor 3). All TQ data.
**Model 6**	This Model adds to (Model 5) school level affluence (factor 1 – LEA data), and the interaction between factor 1 and poor quality of relationships.

Data on thirteen school level variables had been collected at the outset of the trial to facilitate balanced randomization of schools. [24,25] A further two variables related to each other, namely teacher-assessed quality of staff-staff and staff-pupil relationships and these were collected during the trial. Many of the variables were highly correlated, being different measures of the same dimension (e.g. 7 items reflecting school level deprivation). Introducing highly correlated items into regression analysis creates problems of multi-colinearity. To overcome this problem, principal components factor analysis was carried out to reduce the dimensionality (the results of this factor analysis are described in the results section). For each resultant factor (see Results) the factor score was saved and entered into the regression modelling.

In this paper, two-level logistic regression models with pupils at level one and schools at level two were used, and computations were carried out using the MLwiN Version 2.02. Models were estimated using MCMC in MLwiN. Estimates and credible intervals reported in the results are based on a chain of length 10,000 following a burn-in of 1,000. Burn in period and chain length for MCMC estimation varies from one dataset (or even one model) to another. The burn in period should reach stationarity (that is starting off with estimates that are near enough the estimates that you will end up with). The Brooks-Draper and Raftery-Lewis diagnostic confirmed our chain lengths were appropriate. [26-28]

School level variables were incorporated in the modelling to ascertain whether they helped to explain school effects.

Each variable was tested for its interaction with gender to ascertain whether there were any important gender interactions. This was important, as data for male and female pupils were included in the same analysis to optimise power. Nonetheless, school effects were modelled separately for each gender. Modelling was conducted in stages, adding groups of individual level variables to a basic model. The models are described in Table 3.

#### Adjustment for baseline smoking

We explored both adjustment and non-adjustment for baseline smoking. The advantage of adjustment is that it takes account of one of the strongest predictors of current smoking. The disadvantage of adjustment in this study is that by the time of baseline data collection the pupils had been in secondary school for over two years, and thus were already under the influence of any school effect.

#### Early school leavers

Twenty-seven percent of pupils left school at the earliest possible age (leavers) and only 39% of them returned postal questionnaires. Since leavers are more likely to be smokers, there was a danger that schools with more leavers might be advantaged in terms of school effects. The authors addressed this potential bias in four ways.

First, the raw (unadjusted) smoking rates for both ages (14 and 16 years) by early school leaving status at age 16 are presented. We tested whether early leavers who participated in the follow-up at age 16 had a significantly different rate of smoking at age 14 from early leavers who dropped out of the study. There was no significant difference (see Results). This indicates that weighting to compensate for biases created by the leavers who dropped out is an appropriate strategy.

Second, the statistical modelling was conducted using weighted data (using the standardized weight function in MLwiN) to compensate for pupils missing at follow-up, and thus allow inferences applicable to the whole, original sample. The weighting was able to draw on information provided by the subsequent early leavers at baseline, when almost all of them completed baseline questionnaires (96% of the eligible sample, excluding School 4 where no baseline data was collected). The weighted analysis assumes that data are missing at random, [29] conditional on the variables used to calculate the weights. Baseline data plus an indicator of early school leaving (overwhelmingly leaving school for good) were used to develop a predictor of whether a pupil would participate at follow-up. The variables included in the weighting were: parental monitoring, family composition, spending money, early school leaving, sex (male/female), social class and level of alcohol consumption. This predictor was then used to calculate an inverse probability weight in order to estimate responses that would have been provided by pupils had they all participated at follow-up. The inverse probability weights ranged from 1.01 to 9.03, mean of 1.36, and standard deviation of 0.53. Information for those responders in follow-up was used in the same modelling approach described earlier, with the data weighted using the relevant adjustment for each individual. Thus the school-level predictions arising from each model can be thought of as the proportion of current smokers of each gender, adjusted to the levels that would be expected had the non-responding pupils been surveyed. In the results section we will refer to this as the weighted analysis.

Third, we ran the school effects analyses separately for those that that stayed on at school and the leavers who returned questionnaires. Whilst we expect the leavers generally to smoke more than their counterparts at school, this analysis shows whether the school effects are the same for both groups.

Fourth, we allowed the coefficient of 'leaver' at the individual level to be random at the school level. This allowed for the different rates of early school leaving between schools to be taken into account in the modelling. Although not the focus of the paper, this analysis will indicate if there is a school effect on early school leaving.

#### Qualitative data on academic focus

A Spearman's rho correlation between the school's rank in the school effects analysis and the rating for academic focus is presented (see Results). Schools that added value regarding the smoking rate after adjustment had the lowest rank (e.g. 1) and those that lost value after adjustment had the highest rank (e.g. 24).

#### Area effect

We entered information about Local Education Authority geographical location into the model to test for an area effect.

## Results

Overall 25% of male pupils and 39% of female pupils aged 15–16 smoked, with between-school ranges of 8% to 33% for male pupils and 28% to 49% for female pupils.

Table 4 shows that Model 1 identified a number of variables associated with higher rates of smoking at follow-up (age 16), namely: being female; leaving school early; not living with both parents; low levels of parental monitoring; high level of spending money; and being of white ethnic origin (all collected at baseline, except for leaving school early which was coded up at follow-up and based on actual leaving status). Model 2 shows that low levels of both religiosity and self-esteem were associated with higher levels of smoking. Model 3 shows that giving poorer ratings of both attitudes to school and teacher-pupil relationships were associated with increased smoking.

**Table 4:** Odds of being smoker (significant results bolded) estimated from multilevel models

	**Model 1**	**Model 2**	**Model 3**	**Model 4**	**Model 5**	**Model 6**
	Odds (95% CI)	Odds (95% CI)	Odds (95% CI)	Odds (95% CI)	Odds (95% CI)	Odds (95% CI)
Male vs^1 ^female	**0.53**(0.43,0.59)	**0.53**(0.44,0.58)	**0.51**(0.42,0.57)	**0.51**(0.42,0.57)	**0.51**(0.42,0.56)	**0.51**(0.42,0.57)
Age at time of interview	1.00(0.9,1.1)	N/A	N/A	N/A	N/A	N/A
Cohort2 vs cohort 1	0.95(0.79,1.01)	N/A	N/A	N/A	N/A	N/A
Leavers vs non leavers	**1.93**(1.65,2.08)	**1.87**(1.6,2.02)	**1.64**(1.41,1.78)	**1.67**(143,1.81)	**1.65**(1.41,1.78)	**1.67**(1.43,1.81)
Lives with mother only vs both parents	**1.49**(1.24,1.64)	**1.49**(1.24,1.63)	**1.42**(1.18,1.56)	**1.43**(1.19,1.57)	**1.42**(1.19,1.56)	**1.44**(1.20,1.58)
Father only vs both parents	**1.72**(1.24,2.04)	**1.63**(1.18,1.92)	**1.60**(1.16,1.90)	**1.61**(1.16,1.90)	**1.62**(1.17,1.92)	**1.63**(1.18,1.93)
Neither parent vs both parents	**1.9**(1.17,2.42)	**1.92**(1.15,2.50)	**1.72**(1.02,2.24)	**1.75**(1.03,2.28)	**1.72**(1.02,2.24)	**1.75**(1.04,2.28)
Male*mother only vs female*not mother only	**0.73**(0.55,0.84)	**0.73**(0.55,0.85)	**0.76**(0.57,0.88)	**0.76**(0.57,0.88)	**0.76**(0.57,0.88)	**0.76**(0.57,0.88)
Council/Local Authority housing^2 ^vs privately owned housing	1.01(0.86,1.1)	N/A	N/A	N/A	N/A	N/A
Low parental monitoring vs high parental monitoring	**1.46**(1.26,1.57)	**1.40**(1.21,1.51)	**1.20**(1.03,1.3)	**1.20**(1.03,1.29)	**1.19(1.03,1.29)**	**1.20**(1.03,1.30)
Father non-manual vs manual worker	0.99(0.86,1.07)	N/A	N/A	N/A	N/A	N/A
High vs low spending money	**1.29**(1.14,1.37)	**1.27**(1.13,1.36)	**1.20**(1.10,1.31)	**1.24**(1.09,1.33)	**1.23**(1.08,1.31)	**1.25**(1.10,1.33)
Indian subcontinent vs white	**0.50**(0.29,0.67)	**0.54**(0.31,0.71)	**0.48**(0.28,0.64)	**0.48**(0.28,0.63)	**0.48**(0.28,0.64)	**0.49**(0.28,0.66)
Other ethnic group vs white	0.85(0.56,1.05)	0.89(0.58,1.10)	0.87(0.57,1.08)	0.88(0.57,0.09)	0.87(0.57,1.09)	0.90(0.58,1.12)
young mother < 40 vs older mother	1.09(0.95,1.17)	N/A	N/A	N/A	N/A	N/A
Religious belief (higher score less religious)		**1.17**(1.10,1.21)	**1.11**(1.04,1.14)	**1.11**(1.04,1.15)	**1.11**(1.04,1.14)	**1.11**(1.04,1.14)
Self-esteem (higher score lower self-esteem)		**1.17**(1.08,1.22)	**1.11**(1.02,1.16)	**1.11**(1.02,1.16)	**1.11**(1.02,1.16)	**1.11**(1.02,1.16)
Attitude to school (higher score poorer attitudes)			**1.45**(1.34,1.51)	**1.46**(1.35,1.52)	**1.46**(1.35,1.52)	**1.45**(1.35,1.51)
Teacher-pupil relationships (higher score poorer relationships)			**1.13**(1.04,1.17)	**1.12**(1.04,1.17)	**1.13**(1.04,1.17)	**1.13**(1.04,1.17)
School level affluence (higher score means higher affluence)				**1.15**(1.04,1.21)	N/A	**1.27**(1.17,1.32)
School level poor relationships (higher score means poorer relationships)					0.94(0.84,1.01)	0.95(0.85,1.01)
Interaction between school level affluence and relationships						**1.16**(1.05,1.22)

^1'^vs' stands for 'versus the reference category'. Thus for males vs females the odds presented are for males in reference to/compared with females.

^2 ^Council/Local Authority housing is housing rented at an accessible cost from the local government body – normally allocation prioritised based on social need).

The principal components factor analysis to reduce the dimensionality of school level data (see Statistical Methods) identified four factors with eigenvalues greater than 1, accounting for 84% of the variability (see also Table 5). In an earlier factor analysis a variable 'school ethos' loaded only marginally higher (-0.432) with Factor 4 (school size and quality of sex education), but it loaded similarly (0.375) with Factor 2 (access to clinics and parental placing requests). The variable was seen as contaminant and removed before the factor analysis that led to the factor loadings used in the final analysis (see Table 5). A five factor solution was rejected, as eigenvalues dropped steeply from 1.06 for Factor 4 to 0.58 for Factor 5. In the resulting rotated four factor solution it was found that the 7 deprivation-related variables-unemployment in school catchment area, deprivation score of local area, pupils' post school destination, proportion receiving free school meals, staying-on rates (S4 to S5 and S5 to S6), and attendance rates – were grouped together in the first factor. The higher the score on Factor 1 the higher the affluence and so this factor will be called 'school level affluence.' The second factor was dominated by the variables denoting access to clinics and the number of placement requests for a school (this distinguishes urban from rural areas). Pupil-rated teacher-pupil and teacher-teacher relationships comprised the third factor (the higher the score the poorer the relationships and so this factor will be called 'school level poor quality of relationships') and a proxy for school size and quality of sex education the fourth.

**Table 5:** Factor loadings for the variables included in the factor analysis of school level data

	Factor 1 – school level affluence (eigenvalue = 3.73)	Factor 2 – access to clinics and placing requests (reflects urban from rural areas) (eigenvalue = 1.56)	Factor 3 – quality of relationships (eigenvalue = 1.23)	Factor 4 – school size and quality of school sex education (eigenvalue = 1.06)
Deprivation score of local area (linkage between school catchment area postcodes and Carstairs Index of Deprivation)	**-0.922**	0.044	-0.023	0.248
Employment in school catchment area	**0.892**	-0.144	0.136	-0.250
Staying on rates from Secondary (S)4 to S5	**0.866**	0.232	-0.175	0.051
Pupils' post school destination	**0.842**	0.244	-0.147	0.289
Free school meals	**0.819**	-0.115	-0.313	0.278
School attendance	**0.795**	-0.064	-0.348	-0.172
Staying on rates from S5 to S6	**0.774**	0.357	-0.071	0.386
Access to sexual health services	0.051	**0.880**	-0.081	-0.173
Parental placing requests for their child to attend a school (distinguishes urban from rural)	0.041	**0.822**	-0.035	0.101
Proportion of pupils from ethnic minority groups	-0.070	**-0.818**	0.087	0.127
Quality of teacher relationships with each other	-0.028	-0.116	**0.873**	-0.091
Quality of teacher-pupil relationships	-0.334	-0.059	**0.748**	0.059
Size of the school (total number of pupils in S3 & S4)	-0.055	0.184	0.156	**0.803**
Quality of sex education at the school	-0.064	0.515	0.083	**-0.781**

Two factors proved to be significant in explaining school level residuals in smoking, namely Factor 1, school level affluence (highest loadings), and Factor 3, school level (poor) quality of relationships, although Factor 3 was significant only in interaction with Factor 1. These two factors were included as school-level components in subsequent models (see below).

Model 4 (see also Table 4) indicates that, at the school level, higher affluence was associated with higher rates of smoking and Model 5 shows that, at school level, poor quality of relationships was not significant, this is probably because quality of relationships is already adjusted for at the pupil level. However, Model 6 shows that there is an interaction between the school level higher affluence and poor relationships, such that, smoking rates are higher in schools with both higher affluence (and higher attendance and staying on rates) and poor relationships. When this interaction term is fitted, school-level teacher-rated poor relationships was still insignificant on its own, but the effect of school-level higher affluence retained its significance.

School variance parameters were produced as part of the output for each of the models investigated. Sizes of between-school variances relative to Model 1 were of interest here, and percentages in brackets are the proportion of each figure relative to its relevant Model 1 result (Table 6).

**Table 6:** School effects and models to explain these effects (the models are those described fully in Table 3)^1^

	Boys'			Girls'		
	Between-school variance^2 ^(standard error)	95% credible intervals ^3 & 4^	% of model 1 variance^5^	Between-school variance (standard error)	95% credible intervals^4^	% of model 1 variance
**Model 1 **– adjusted for socio-demographic and cultural factors predicting smoking.	**0.250 **(0.099)	(0.114,0.497)	(100%)	**0.046 **(0.021)	(0.012,0.081)	(100%)
**Model 2 **– adds (to model 1) individual cognition measures.	**0.275 **(0.111)	(0.122,0.551)	(110%)	**0.048 **(0.023)	(0.015,0.112)	(104%)
**Model 3 **– adds (to model 2) cognitions relating to school.	**0.240 **(0.105)	(0.116,0.520)	(96%)	**0.030 **(0.014)	(0.011.0.067)	(65%)
**Model 4 **– adds (to model 3) school level affluence (factor 1).	**0.175 **(0.077)	(0.068,0.302)	(70%)	0.040 (0.024)	(0.011,0.101)	(86%)
**Model 5 **– adds (to model 3) school level poor quality of relationships – factor 3	**0.201 **(0.084)	(0.088,0.408)	(80%)	0.038 (0.021)	(0.011,0.91)	(83%)
**Model 6 **– adds (to model 5) school level affluence (factor 1), and the interaction between factor 1 and poor quality of relationships.	0.045 (0.023)	(-0.001,0.112)	(18%)	0.039 (0.020)	(0.000,0.077)	(85%)

^1^Statistically significant results are bolded

^2 ^Between-school variance is the difference between the average value of smoking at school level (based on the data i.e. actual smoking of pupils in a school) and the estimates obtained from the modelling. The aim is to develop models whereby the actual school level smoking values are close to the estimated values, thus lowering the variance is what is desired.

^3 ^The Credible Intervals (produced as part for the MCMC estimation procedure) represent the difference in an outcome between a school at the bottom (2.5^th ^centile) and the top (97.5^th ^centile) of the distribution.

^4 ^Results are significant at the alpha = 0.05 level when the 95% credible intervals for the between school variance do not include zero.

^5 ^As described in the Introduction, a 'school effect' is when 'pupil outcomes for a school vary, either positively or negatively, from that which might be expected, given the known predictors of these outcomes' (i.e. between-school variance after adjusting for Model 1 predictors). Given that Model 1 adjusts for the known predictors of smoking, the results for Model 1 indicate that there is a significant 'school effect' on males' and females' smoking behaviour at age 16. Model 6 has successfully explained that 'school effect' for males, while Model 3 explained the 'school effect' for girls.

Table 6 shows that there is a large significant school effect for males and a smaller but still significant school effect for females when socio-economic and cultural factors (compositional factors) are taken in to account.

The addition of individual cognition measures (self-esteem and religiosity – Model 2) slightly increased school level variance for both male and female pupils. The introduction of attitude towards school and pupil rated quality of staff-pupil relationships (Model 3) decreased the school variance for male pupils by 4% (from it's original level) and decreased it by 35% for female pupils, at this point the school level variance becomes insignificant for females, but remains significant for males. The introduction of the school level Factor 1, higher affluence and staying on rates and attendance (Model 4), decreased the variance explained to 70% of its original level for males. Introducing teacher-assessed (poor) quality of relationships (Factor 3), on its own, that is without Factor 1, reduces variance to 80% for males, but this is not as large a reduction as that observed for Factor 1. Finally, when both school level deprivation and school level (poor) quality of relationships are entered together and their significant interaction fitted (Model 6), the male pupils' school-level variance plummets to 18% of the original. After fitting Model 6 the observed school effect for male pupils became insignificant, that is, it has been explained.

**Figure 3:**
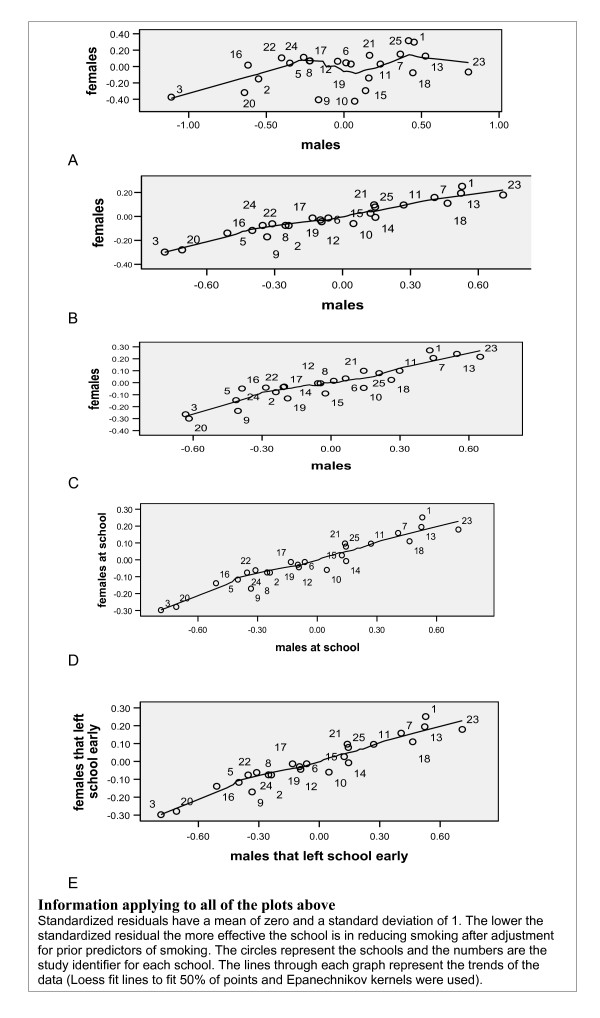
**A: Plot showing standardized school level residuals (school effects) for males versus females BEFORE adjusting for known predictors of smoking (NULL MODEL).** B: Plot showing standardized school level residuals (school effects) for males versus females AFTER adjusting for known predictors of smoking (Model 1, see Table 3). C: Plot showing standardized school level residuals (school effects) for males versus females after adjusting for known predictors of smoking (Model 1, see Table 3) and PRIOR SMOKING. D: Plot showing standardized school level residuals (school effects) for male versus female PUPILS STILL AT SCHOOL after adjusting for known predictors of smoking (Model 1, see Table 3). E: Plot showing standardized school level residuals (school effects) for male versus female PUPILS THAT LEFT SCHOOL EARLY after adjusting for known predictors of smoking (Model 1, see Table 3).

Figure 3A illustrates the school effect before adjustment (null model), it is clear that there is variance to be explained and the schools are very scattered. Figure 3B shows that, once individual compositional factors have been taken into account, the plot becomes more linear and the variance has decreased, reflecting the power of the adjustment for pupil composition in explaining the variance in the raw smoking rates. There is a positive correlation between the schools that leads to lower smoking rates for male pupils and higher rates for female pupils. The size of the school effect was much smaller for females than for males. We continued to explore data on school characteristics separately for males and females.

### Adjustment for baseline smoking

There is a highly significant correlation (r = 0.95, p < 0.000 for males and r = 0.85, p < 0.000 for females) between the standardised residuals (school effects) for Model 1 when baseline smoking is adjusted for and when it is not (This is further illustrated by comparing Figure 3B and Figure 3C). As the findings will be the same whether we adjust for baseline smoking or not, and given our concerns about the pupils being already under the influence of the school at baseline, we decided to continue the modelling unadjusted for baseline smoking. The school effect for onset of smoking between S3 (age 14) and S5 (age 16) is almost identical to the school effect for smoking at S5. This shows robustness in the school effect.

### Early school leavers

Table 7 shows that, for both sexes, there is a very similar rate of smoking at age 14 between leavers that returned postal questionnaires at age 16 and leavers that did not return a postal questionnaire. A t-test showed no significant difference between the two groups for males and females (t = 0.269, df = 23, p = 0.790 and t = 0.819, df = 23, p = 0.421 respectively). As expected, for both sexes, pupils that stayed on at school had a significantly lower rate of smoking than early leavers (results not presented).

**Table 7:** School characteristics and leavers' questionnaire response – smoking rates for 'stayers on' and leavers (by whether they returned a postal questionnaire at age 16)

**School characteristics and leavers' questionnaire response**				**Smokers (%)**									
				**Stayed on at school after age 16**				**School leavers who returned age 16 postal questionnaires**				**Leavers who did NOT returned age 16 postal questionnaires**^1^	
				
				**age 14**		**age 16**		**age 14**		**age 16**		**age 14**	
**School**	**Eligible sample N**	**% that left school early**	**% of early school leavers returning questionnaire age 16 data**	**boys**	**girls**	**boys**	**girls**	**boys**	**girls**	**boys**	**girls**	**boys**	**girls**

1	321	20.9	38.8	16.7	24.8	30.1	48.3	40.0	26.7	71.4	45.5	35.7	22.2
2	115	18.3	33.3	14.0	20.5	11.1	33.3	100.0	66.7	100.0	66.7	0.0	0.0
3	209	36.4	43.3	5.1	22.2	8.1	25.0	10.0	11.2	0.0	40.0	21.1	26.7
4	380	18.4	47.1	N/A*	N/A*	29.2	45.5	N/A*	N/A*	40.0	52.9	N/A*	N/A*
5	401	36.4	39.0	15.6	29.8	11.2	37.0	43.8	58.3	45.4	58.3	31.1	38.5
6	327	31.2	28.4	12.9	27.8	23.1	40.7	15.4	23.1	33.3	41.7	30.6	45.5
7	306	25.2	33.3	12.6	21.9	30.1	41.0	50.0	60.0	50.0	61.5	54.5	57.1
8	292	48.6	31.7	4.8	19.0	19.2	35.3	14.3	35.7	12.5	60.9	11.3	38.2
9	301	34.2	40.7	13.8	21.6	16.4	21.1	47.1	52.4	46.2	47.1	47.1	41.7
10	392	17.3	36.8	11.3	16.0	25.2	28.0	0.0	17.6	33.3	38.5	16.7	23.1
11	272	16.5	35.5	12.1	27.9	25.3	37.5	14.3	71.4	66.7	75.0	46.7	58.3
12	371	29.9	36.0	11.0	27.9	13.8	36.5	36.4	42.1	85.7	62.5	22.5	44.4
13	510	21.0	39.2	13.3	18.8	33.1	40.1	22.2	61.9	50.0	55.6	28.9	30.0
14	316	16.8	52.8	21.0	11.9	28.1	31.5	23.1	46.7	12.5	33.3	36.4	30.0
15	380	15.8	43.3	15.7	31.0	26.6	33.6	28.6	58.3	28.6	50.0	35.3	58.3
16	376	34.0	41.4	5.6	15.1	11.5	38.5	50.0	30.3	28.6	48.1	7.7	36.8
17	264	28.4	29.3	15.3	25.9	16.9	37.8	0.0	27.3	50.0	77.8	41.7	71.4
18	397	23.7	37.2	21.3	28.2	33.1	34.9	16.7	52.4	25.0	50.0	27.6	40.9
19	303	36.0	41.3	19.8	35.2	20.0	33.8	46.2	57.1	37.5	57.9	20.8	43.3
20	370	39.5	42.5	4.6	25.7	12.9	26.7	12.5	36.6	16.7	40.7	26.7	46.9
21	559	21.6	46.3	13.0	27.7	25.5	40.9	20.8	57.1	37.5	60.0	34.2	36.8
22	306	25.8	35.4	16.8	29.4	13.6	41.2	35.7	63.6	28.6	66.7	30.0	52.6
23	293	15.7	30.4	19.5	16.3	39.2	34.3	40.0	44.4	50.0	71.4	33.3	53.3
24	397	32.5	49.6	8.1	26.2	17.9	36.4	26.7	38.1	40.0	82.4	18.2	54.2
25	272	50.7	48.6	10.0	33.3	25.9	50.0	24.0	36.8	41.2	46.7	23.7	40.9
**All schools**	**8430**	**27.4**	**39.2**	**13.6**	**24.0**	**23.9**	**36.8**	**27.2**	**43.5**	**38.8**	**53.9**	**27.3**	**43.2**

* N/A as School 4 withdrew from the baseline survey and thus no data can be provided (see also Methods)

^1 ^Age 16 values could not be observed for those who did not return the postal questionnaire.

It is reassuring that the school effect looks robust when analysed separately for non-leavers and leavers (Figures 3D &3E). This is further substantiated by a highly significant correlation between the two groups (r = 0.95, p < 0.000 for males and r = 0.86, p < 0.000 for females). The key point of this finding is that attrition at follow-up is not responsible for causing the variation across schools and that the school effect findings remain stable when analysing the leavers and non leavers together, especially given the weighting.

### Qualitative data on academic focus

**Figure 4:**
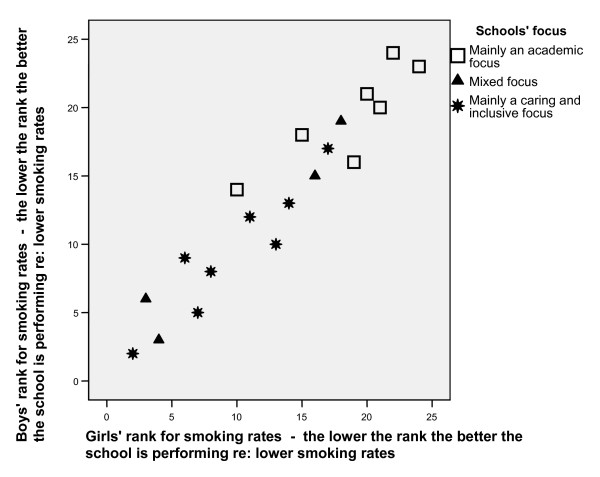
Schools' main focus by boys' and girls' rank for school level smoking rates.

For both male and female pupils, a significant Spearman's rho correlation was found between schools' rank in the school effects analysis and the qualitative researchers' rating of schools' academic focus (r = -0.716, p = 0.001 for boys and r = -0.657, p = 0.002 for girls). Figure 4 illustrates that the schools that had the lowest rates of smoking after adjustment (i.e. schools that added value regarding their smoking rate) tended to be the schools that had their emphasis on caring and inclusiveness rather than solely on an academic focus.

### Area effects

When information on Local Education Authority (geographical) location was entered in the model it was insignificant (results not shown). Thus after adjustment for compositional factors there is no evidence of an area effect at this level.

## Discussion

The rates of smoking found in this study (25% for male pupils and 39% for female pupils) reflect prevalence figures reported elsewhere, both by gender and for this age group, from similar samples. [19,30]

This study included a wide range of individual level socio-economic and cultural characteristics, in order to establish that any school effect was not attributable to different pupil composition between schools. It was not our intention to focus on these characteristics. However, it is of note that among these factors the most powerful predictors of smoking are (in order of effect magnitude): baseline smoking (not presented); leaving school as early as legally possible; not living with both parents; low levels of parental monitoring; being female; having a high level of personal spending money; and being white rather than Indian or Pakistani. These results confirm previous models of individual predictors of adolescent smoking [19].

Two central questions were addressed in this paper: (i) are there differences between schools in smoking rates, once socio-demographic and individual predictors are taken into account? and (ii) can any such differences be explained by school characteristics? We have shown that, amongst a representative sample of Scottish pupils aged 15/16, there is a large amount of school variance in smoking rates amongst males, and a smaller amount of school variance amongst female pupils. For both genders this variance remains statistically significant after controlling for known predictors.

There are a number of limitations to this study mainly because the data was collected for a cluster randomised trial of teacher-delivered sex education [20] and not purposively to study school effects on smoking. We would have preferred to have adjusted for late primary school smoking, as per West et al. (2004). However, West et al. found that while prior primary school smoking was highly predictive of follow-up smoking, the adjustment had only a modest impact on the school effect, so it may not have made a substantive difference to the story of this paper. We did adjust for our rather later baseline smoking (at age 14) and the school effects were robust with or without that adjustment.

We did not collect data on school-based smoking education or school level smoking policies. A recent Cochrane review [8] concluded that school-based smoking education is largely ineffective, and so collecting such data might not have influenced the results substantially. It is not clear whether data on school level smoking policies would have affected the results: this would be a very interesting avenue for future research.

Since follow-up data were collected when many pupils were aged over 16, the minimum school leaving age in Britain, 27% of the pupils had already left school. In our analysis we were careful to address the possible bias this might have introduced. First, as expected, early leavers have a higher rate of smoking than those that stayed on at school and we have adjusted for that in our modelling. Related to this point, we demonstrated that for early school leavers there was no significant difference in smoking rates at age 14 between those that returned a postal questionnaire and those that did not. Under the missing at random assumptions (MAR) this confirms that our weighting strategy would fairly compensate for the pupils missing at follow-up. More information on MAR is available from James Carpenter and Mike Kenwood's website on 'Missing Data'. [29] Second, as schools have different rates of early leavers, we allowed leavers to vary randomly at the school level in our modelling in order to adjust for this issue. A secondary finding, which resulted from this modelling strategy, was that the school effect on smoking was not restricted to those staying on at school but also included early leavers. This effect was attenuated when attitude to school and perceived quality of teacher-pupil relationships were taken into account. Third, we also ran the models separately for leavers and non-leavers and demonstrated that the school effect for both groups was very highly correlated. Thus, the finding of school effects on smoking and the factors that help explain it are robust across leavers and those that stayed on at school.

Another limitation is related to the issue of multiple (statistical) comparisons. The questions we were aiming to answer in this paper were complex in nature, and we needed to check a lot of different combinations of factors to explore the issues. The danger of multiple comparisons is that some of the findings could be spurious. However, it may be worth noting that the analysis in this paper was theoretically based and the findings make sense within the theoretical framework. In addition, the findings that simply identified the predictors of smoking within this dataset replicate previous findings [19] and the more complex modelling to establish and explain school effects on smoking replicate the findings of previous findings, albeit with the added dimension of gender. [17-19] Replication is a strategy for recognising real findings from spurious findings, thus the fact that the findings in this paper replicate those from previous studies is reassuring. Future research will further address the issue of multiple comparisons.

West et al. found smaller school effects with increasing age of school pupils, [19] but they did not run the analysis separately by gender. It would be interesting for future research to establish how the school effects change with age for both genders, as it may be that males and females are affected by school differentially at different ages and unpacking this could help influence future interventions.

The study found that pupil rated teacher-pupil relationships and attitude to school both attenuated the school-level variance for both genders. Furthermore, the qualitative researchers' data further supported this finding by showing that schools rated as caring and inclusive had lower rates of smoking for both male and female pupils. Thus, quality of relationships within schools has a measurable impact on school pupils' smoking rates. These findings echo those of West et al., though they were based on younger age groups, and they provide support for a Health Promoting School approach. [6,31]

A counter-intuitive finding was the interaction between school-level affluence and school-level poor quality relationships, such that schools with high affluence, but that are reported to have poor quality relationships, had higher levels of smoking for males. When this interaction is fitted the school effect for males was dramatically attenuated. This result should not detract from the findings presented above. It suggests, perhaps surprisingly, that the effect of poor teacher-pupil relationships on smoking is greater in schools which are affluent, after adjusting for affluence in their individual pupils. Thus, we are describing the impact of the school effect on a pupil attending an affluent school who is 'average' in terms of the whole sample. The affluent schools, particularly those with poor relationships, may be more likely than deprived schools to have an academic focus, perhaps at the cost of the social climate or health related goals. This explanation would be compatible with the findings of a qualitative study that concluded that pursuing health and education objectives involved competing values and priorities which could create tensions in the context of limited resources. [32] However, this area is complex and other research has suggested that it is possible for schools to achieve both academic and health goals, depending on the approach they take. [33,34]. However, there is a need for further, particularly qualitative, work to understand this finding more fully.

This study successfully explained the school-level variance for both male and female pupils. This was despite the fact that the data were collected to evaluate sex education, not to explain school effects on smoking. There is much scope for future studies purposively designed to assess the importance of a wider range of school features [18].

This study did not detect a significant local education authority geographical area effect. This is compatible with previous research which has indicated that the amount of variance attributable to school effects is larger than that attributable to area or neighbourhood effects for smoking. [19,35] Nonetheless, purposive future research could explore small area effects that were beyond the scope of this study.

## Conclusion

We observed 'school effects' on rates of smoking for males and, to a lesser extent, females at 15/16 years. For male school pupils, attitude to school, quality of staff-pupil relationships, school-level affluence and its interaction with school level poor quality of staff-pupil relationships, were all associated with school level smoking rates and successfully explained the 'school effects'. It is likely that there are additional effects of peer influence and perhaps small geographical area effects. However, peer influence is itself subject to the school effects and evidence to date suggests that area effects are smaller than school effects. Therefore, our results suggest that changing school characteristics may have an effect on smoking and so support a Health Promoting School approach. These findings emphasise that investment in social environments has the potential to strongly influence male smoking and to a lesser extent female smoking. This influence is possible even for senior secondary school pupils and is likely to extend to other health behaviours.

## Competing interests

The authors declare that they have no competing interests.

## Authors' contributions

MH, DW and CA designed the original study, while MH and DW collected the data. MH and RE analysed the data. CA and DW commented on the analysis. MH and RE drafted the paper and MH and DW revised subsequent drafts based on co-authors' comments. MH, RE, DW and CA commented on subsequent drafts of the paper.

## Pre-publication history

The pre-publication history for this paper can be accessed here:


